# The rise and fall of basal bodies in the nematode *Caenorhabditis elegans*

**DOI:** 10.1186/s13630-017-0053-9

**Published:** 2017-07-26

**Authors:** Inna V. Nechipurenko, Piali Sengupta

**Affiliations:** Department of Biology and National Center for Behavioral Genomics, Brandeis University, Waltham, MA 02454 USA

**Keywords:** *C. elegans*, Sensory neurons, Centriole, Basal body, Degeneration, Central tube, Diverse cilia morphologies, Ultrastructure

## Abstract

The free-living nematode, *Caenorhabditis elegans*, is a widely used genetic model organism for investigations into centriole and cilia biology. Only sensory neurons are ciliated in *C. elegans*; morphologically diverse cilia in these neurons are nucleated by basal bodies located at the dendritic endings. *C. elegans* centrioles comprise a central tube with a symmetric array of nine singlet microtubules. These singlet microtubules remodel in a subset of sensory neurons to form the doublet microtubules of the basal bodies. Following initiation of ciliogenesis, the central tube, but not the outer centriole wall, of the basal body degenerates. Recent ultrastructural characterization of basal body architecture and remodeling have laid the foundation for future studies into mechanisms underlying different aspects of basal body genesis, remodeling, and intracellular positioning.

## *Caenorhabditis elegans:* the organism

The genus *Caenorhabditis* belongs to the diverse order of Rhabditida that includes free-living as well as parasitic species within the phylum Nematoda and superphylum Ecdysozoa. The model organism, *Caenorhabditis elegans*, is a free-living bacteriovorous nematode that is found in multiple geographical locations [1]. Although *C. elegans* was originally identified in compost heaps [2], these nematodes are also found in association with rotting fruits and other decomposing plant material [1, 3–5]. The commonly used N2 laboratory reference strain was originally isolated in Bristol, England, in 1951 but was passaged for many generations before being frozen [6]. As a consequence, this strain contains multiple fixed alleles that are likely to be adaptive for laboratory growth conditions [6]. Related free-living species that are also studied experimentally include *Caenorhabditis briggsae, Caenorhabditis remanei*, *Caenorhabditis brenneri*, and *Caenorhabditis japonica* [4, 7].

### Diverse cilia in *C. elegans*

**Fig. 1:**
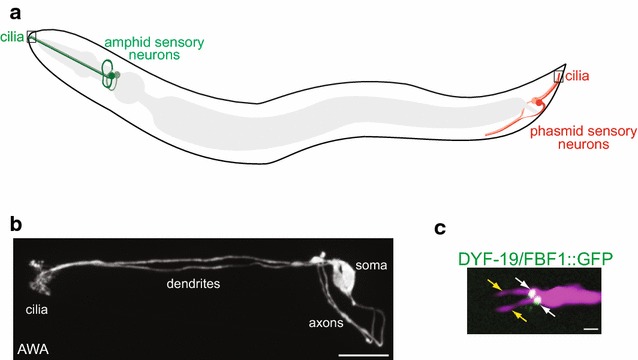
Ciliated sensory neurons in *C. elegans*. **a** Diagram (not to scale) of a *C. elegans* hermaphrodite showing a subset of the amphid (*head*) and phasmid (*tail*) ciliated sensory neurons (*green* and *red* cells, respectively). The intestine and pharynx (*gray*) are also shown. **b** A confocal image of the amphid AWA sensory neuron pair in an adult hermaphrodite. Anterior is to the left. Note the extensively branched cilia of this neuron type. Image courtesy: Ashish K. Maurya. **c** A confocal image showing two cilia (*yellow arrows*) present at the dendritic ending of an amphid AWB neuron in the adult hermaphrodite. The GFP-tagged DYF-19/FBF1 transition fiber protein (*white arrows*) is localized to the base of the cilia. Anterior is at left. *Scale bars* in **b** and **c** 10 and 1 μm, respectively

*Caenorhabditis elegans* populations contain both hermaphrodites and males. 60 ciliated cells are present in common in adult hermaphrodites and males, but males also contain additional ciliated cells [8–12]. All ciliated cells in both males and hermaphrodites are postmitotic sensory neurons, with the majority of male-specific ciliated neurons implicated in the regulation of mating behaviors [13, 14]. All cilia in *C. elegans* are non-motile and located at the dendritic endings of sensory neurons (Fig. 1a–c). These cilia are remarkably diverse in their appearance and ultrastructure, ranging from simple rod-like structures to cilia exhibiting highly branched, curved, or large fan-like morphologies [8, 9, 15, 16] (Fig. 1b, c). Underlying axonemal structures are also diverse and are built via neuron-specific deployment of tubulin isotypes, intraflagellar transport mechanisms, and ciliary trafficking pathways (e.g. [16–22]). The diversity of cilia structures, and the conservation of many ciliary and centriole/basal body genes in *C. elegans*, together with the fact that cilia are not essential for its viability, have made this organism an invaluable model for investigations into centriole and cilia biology.

## Basal body origins and structure

### Centriole structure

**Fig. 2:**
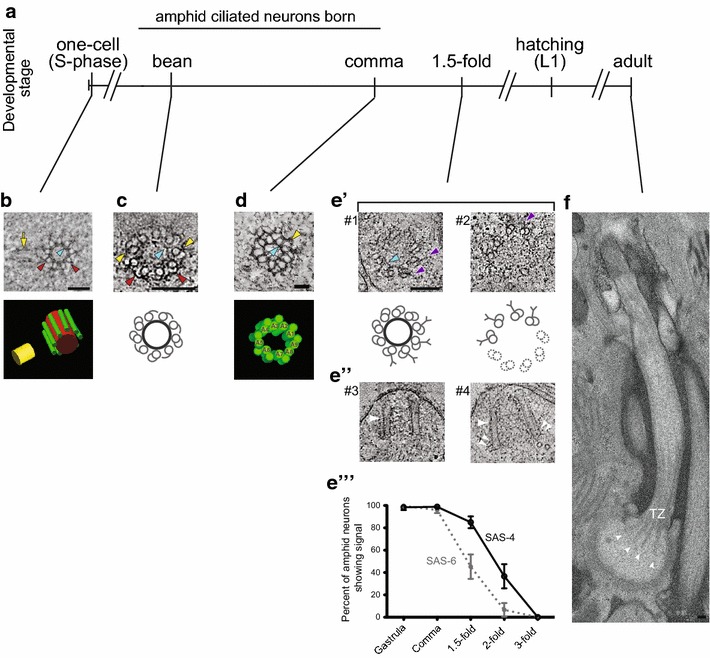
Ultrastructure of *C. elegans* centrioles and basal bodies. **a** Developmental timeline of *C. elegans* and time of birth of amphid sensory neurons. **b**–**e′** Representative cross-section tomographic slices and three-dimensional models or cartoons showing centrioles in the one-cell embryo and basal bodies in amphid neurons at the indicated developmental stages. Note basal bodies containing (*#1*) or lacking (*#2*) a central tube in **e′**. Central tube, sMTs, and the daughter centriole are indicated in *red*, *green* and *yellow*, respectively in the model in **b**. A1–A9 indicate dMTs in **d**. *Red arrowheads*: sMTs with hooks, *yellow arrowheads*: dMTs, *blue arrowheads*: central tube, *purple arrowheads*: Y-links of the transition zone, *yellow arrow*: daughter centriole. **e′′** Longitudinal tomographic slices showing basal bodies/axonemes. Large *white arrowheads*: basal bodies/axonemes, *small white double arrowheads*: flared dMTs at the base. **e′′′** Quantification of SAS-6 and SAS-4 signals at the amphid sensory neuron basal bodies through embryonic development. **f** A longitudinal section of the amphid ASE neuron cilium in the adult hermaphrodite. Note flared dMTs at cilia base (*arrowheads*). *TZ* transition zone. Images in **b** are adapted by permission from Macmillan Publishers Ltd: [24]. Images in **c**, **e′** and **e′′** are adapted from [25]. Images in **d** and **e′′′** are adapted from [26]. Image in **f** is adapted from [15].* Scale bars* **b**, **c**, **e′**–**e′′′**, **f** 100 nm, **d** 50 nm

*Caenorhabditis elegans* centrioles are typically smaller than vertebrate centrioles, and are approximately ~100 nm in width and ~100–180 nm in length [23–25]. These centrioles are composed of a radially symmetric array of nine singlet microtubules (sMTs) surrounding a central tube [24–26] (Fig. 2b) which, in a recent study, was shown to contain a cartwheel-like structure similar to that present in centrioles of other organisms [23]. In the early embryo, centriole sMTs appear to be associated with electron-dense ‘appendages’ [24] (Fig. 2a, b), also termed a ‘paddlewheel’ [23] of unknown function. At later embryonic stages, hook-like appendages associated with centriole sMTs close to form the B-tubules of the basal body in a subset of ciliated sensory neurons (see below) [25] (Fig. 2c, d).

### Centriole to basal body conversion and basal body structure

56 of the 60 ciliated sensory neurons shared between hermaphrodites and males are born during embryogenesis, whereas all male-specific ciliated neurons, except for the four cephalic CEM neurons, develop post-embryonically [11, 12]. To date, centriole to basal body conversion has been characterized only in a subset of embryonically generated ciliated sensory neurons of the bilateral amphid organs of the head in the *C. elegans* hermaphrodite [25, 26] (Fig. 1a).

*Caenorhabditis elegans* embryogenesis follows a temporally stereotyped progression with an early proliferative stage followed by a later organogenesis/morphogenesis stage [11]. Following the birth of terminally differentiated amphid sensory neurons at the beginning of the organogenesis/morphogenesis period (Fig. 2a) and initial outgrowth of their dendrites, the dendritic tips are anchored in place at the presumptive nose in the embryo, while the neuronal soma translocate posteriorly [27]. Examination of dendritic endings following the commencement of somal translocation showed the presence of a single centriole/basal body, consistent with the presence of a single cilium in the majority of amphid sensory neurons [25, 26]. However, two centrioles/basal bodies were also observed in a subset of neurons, presumably correlating with two cilia found in each of the remaining three amphid sensory neuron pairs [25, 26] (see Fig. 1c). The origin of the second basal body in these neurons is unknown.

Visualization of centrioles in a subset of amphid sensory neurons immediately following terminal differentiation showed that the hook-like appendages associated with the A-tubules of the centrioles close to form doublet (AB) MTs (dMTs) of the basal body [25, 26] (Fig. 2c, d). This A- to AB-tubule conversion occurs asynchronously, such that both A-tubules with hooks and AB dMTs were observed within the same 70 nm section by electron microscopy [25] (Fig. 2c). These dMTs subsequently grow asynchronously to template the transition zone and the axoneme [25]. Thus, basal bodies in amphid sensory neurons are comprised of a radially symmetric array of nine dMTs surrounding a central tube (Fig. 2d).

It is important to note, however, that since early steps in ciliogenesis have been visualized only in a subset of ciliated sensory neurons, it is unknown whether similar remodeling of centriole sMTs to dMTs accompanies basal body generation in other ciliated neuron types in *C. elegans.* For example, the transition zones of the chemosensory IL2 inner labial neurons contain 5–7 Y-linked dMTs instead of the canonical 9 dMTs [8, 15], raising the possibility that basal bodies in these neurons may exhibit distinct structural features.

### Basal body-associated structures

*Caenorhabditis elegans* basal bodies appear to lack canonical centriole appendages as determined via transmission electron microscopy and serial section electron tomography of high pressure-frozen and freeze-substituted (HPF-FS) embryos [25, 26]. However, electron-dense material of unknown identity has been observed between dMTs and the cell membrane in subciliary regions proximal to the transition zone [26]. In addition, distal appendage/transition fiber components such as DYF-19/FBF1 localize to the basal body region in adult sensory neurons [28, 29] (Fig. 1c). Since mesh-like structures in HPF-FS samples can be less clearly visible than those in chemically fixed specimens [30], these observations raise the possibility that appendage-associated proteins in *C. elegans* ciliated neurons may be organized in structures distinct from those observed in organisms with canonical basal bodies.

Although *C. elegans* basal bodies appear to lack canonical distal and subdistal appendages, transmission electron microscopy has shown that two distinct types of rootlets associate with basal bodies in different classes of sensory neurons. The IL1, OLQ, and BAG sensory neurons have striated rootlets, while amphid neurons contain amorphous rootlet-like material at the cilia base [8, 15, 31]. Basal bodies in amphid sensory neurons are also found in close proximity to apical junctions, cell adhesion complexes between the dendritic membrane and the membranes of surrounding glial support cells [32–34]. It is possible that in the absence of transition fiber-like structures that permit docking of the basal body to the membrane, association of the basal body with the cytoskeleton and apical junctions anchors the basal body at the dendritic tip [34].

### Identification of basal body components

Although no proteomic screens to identify basal body components have been performed in *C. elegans*, a number of genetic and functional genomics screens have led to the identification of key evolutionarily conserved components necessary for centriole assembly (e.g. [35–38]). Since only sensory neurons are ciliated in *C. elegans*, in theory, mutations in basal body genes should be readily isolated in forward genetic screens for viable animals with ciliary defects. Indeed, mutations in *dyf*-*19*/FBF1 were identified in such an unbiased screen [29]. Screens for mutants with ciliogenesis defects are not yet saturated [39], suggesting that additional basal body genes remain to be identified by this approach. *C. elegans* basal body molecules have also been identified via biochemical approaches [40] and by homology-based analyses [41].

## Basal body lifecycle

### Remodeling of basal bodies

A unique feature of *C. elegans* is that basal bodies do not appear to be present in sensory neurons in adults, leading to the notion that basal bodies degenerate in this organism [8]. However, recent studies suggest that while the central tubes of basal bodies in the amphid sensory neurons indeed degenerate during late embryonic development, the dMTs of the outer centriole wall persist throughout development [25, 26]. This model is supported by the observations that central tube components (e.g. SAS-5, SAS-6) are lost in adult ciliated neurons [26, 42]. Interestingly, while a subset of outer centriole wall-associated proteins (e.g. HYLS-1) is retained, other components (e.g. SAS-4) are also lost in adults [28, 40], suggesting that the composition of the outer centriole wall is modified during development. Degeneration of the central tube, and loss of SAS-4 and SAS-6 were noted beginning at the 1.5-fold stage of embryonic development, prior to axoneme extension [25, 26] (Fig. 2e′, e′′′). Degeneration of the central tube correlates with ‘flaring’ of the dMTs of the basal body [25] (Fig. 2e′′); these flared dMTs at the ciliary base in adults (Fig. 2f) were previously erroneously identified as transition fibers [8]. Thus, the centriole core is essential for initiation of ciliogenesis, but is dispensable for axoneme elongation.

### Does the basal body have the function of a centrosome?

All ciliated cells in *C. elegans* are terminally differentiated sensory neurons. The vast majority of dendritic MTs in these neurons are oriented with their minus ends out (i.e. toward the dendritic tips where basal bodies are localized) [43, 44]. In adult amphid neurons, a subset of 11-protofilament dendritic MTs is found in the periciliary membrane compartment at the cilia base [15, 33]. These MTs occasionally enter the proximal-most few hundred nanometers of the dMT region of the remodeled basal body/axoneme; however, they do not appear to be basal body-anchored [15]. Intriguingly, γ-tubulin is present at the base of cilia in the head amphid and tail phasmid sensory neurons of adult *C. elegans* hermaphrodites [45]. Although the role of γ-tubulin in these classes of ciliated neurons is presently unclear, together these findings raise the possibility that basal bodies in *C. elegans* ciliated sensory neurons may function as microtubule organizing centers (MTOCs).

## Notable basal body findings and future prospects

### Basal body findings from *C. elegans*

Although seminal studies in *C. elegans* led to the identification of highly conserved centriole components including the core centriole assembly pathway [46], basal bodies are only beginning to be studied in this organism. Recent work has delineated the series of basal body-associated events that regulate ciliary docking and import of intraflagellar transport (IFT) machinery critical for cilia assembly and maintenance. These studies have shown that the outer centriole wall/basal body component HYLS-1 recruits DYF-19/FBF1, which in turn directly interacts with the IFT-B component DYF-11/IFT54 and facilitates ciliary entry of IFT particles [26, 28, 29]. In another study, the multifunctional conserved protein Girdin was identified in *C. elegans* as playing a major role in positioning the basal body, and shown to play a similar role in ciliated mammalian cells [34]. The recent detailed characterization of the centriole to basal body conversion and remodeling of the basal body following initiation of ciliogenesis [25, 26] now opens up new avenues of research into the underlying mechanisms.

### Strengths and future of basal body research in *C. elegans*

In addition to its genetic tractability and expansive research toolkit, *C. elegans* offers a number of unique advantages for cilia and basal body research. First, as indicated above, the ability to identify viable mutants defective in basal body structure and/or function allows for the identification of new basal body genes. Second, since basal body position determines the site of ciliogenesis and thus, correct cilia function, the localization of basal bodies at the dendritic tips of *C. elegans* sensory neurons provides an attractive experimental system in which to examine mechanisms of basal body positioning. Third, how the central tube is specifically targeted for degradation while largely sparing the outer centriole wall, and whether and how this degeneration is coordinated with developmental age will be exciting areas of study. The degeneration of the central tube is partly reminiscent of the process of centriole elimination observed in gametogenesis in many organisms [47], suggesting that studying this process in *C. elegans* may provide insights into related processes in other systems. Finally, the availability of a subset of basal body markers will now allow investigations into possibly diverse mechanisms of basal body genesis and structure in cells with unique axoneme ultrastructures. We expect that ongoing and future investigations into the biology of the nematode basal body will continue to provide new insights into the structure and function of this important organelle.

## Abbreviations

MTs: microtubules
sMTs: singlet microtubules
dMTs: doublet microtubules
HPF-FS: high pressure-frozen and freeze-substituted
MTOC: microtubule organizing center
